# Peptide-Mediated Tumor Targeting by a Degradable Nano Gene Delivery Vector Based on Pluronic-Modified Polyethylenimine

**DOI:** 10.1186/s11671-016-1337-5

**Published:** 2016-03-01

**Authors:** Zhaoyong Wu, Shuyu Zhan, Wei Fan, Xueying Ding, Xin Wu, Wei Zhang, Yinghua Fu, Yueyan Huang, Xuan Huang, Rubing Chen, Mingjuan Li, Ningyin Xu, Yongxia Zheng, Baoyue Ding

**Affiliations:** Department of Pharmacy, Jiaxing Maternal and Child Health Care Hospital, Affiliated Hospital of Jiaxing University, Jiaxing, People’s Republic of China; Department of Pharmaceutics, Medical College of Jiaxing University, Jiaxing, People’s Republic of China; Department of Pharmacy, The 425th Hospital of PLA, Sanya, People’s Republic of China; Department of Pharmaceutics, Shanghai First People’s Hospital, Shanghai Jiaotong University, Shanghai, People’s Republic of China; Department of Pharmacy, Shanghai Pulmonary Hospital, Shanghai, People’s Republic of China

**Keywords:** Pluronic-PEI, DR5-TAT peptide, Tumor-targeting gene delivery system, Cytotoxicity, Transfection efficiency

## Abstract

Polyethylenimine (PEI) is considered to be a promising non-viral gene delivery vector. To solve the toxicity versus efficacy and tumor-targeting challenges of PEI used as gene delivery vector, we constructed a novel non-viral vector DR5-TAT-modified Pluronic-PEI (Pluronic-PEI-DR5-TAT), which was based on the attachment of low-molecular-weight polyethylenimine (LMW-PEI) to the amphiphilic polymer Pluronic to prepare Pluronic-modified LMW-PEI (Pluronic-PEI). This was then conjugated to a multifunctional peptide containing a cell-penetrating peptide (TAT) and a synthetic peptide that would bind to DR5—a receptor that is overexpressed in cancer cells. The vector showed controlled degradation, favorable DNA condensation and protection performance. The Pluronic-PEI-DR5-TAT/DNA complexes at an N/P ratio of 15:1 were spherical nanoparticles of 122 ± 11.6 nm and a zeta potential of about 22 ± 2.8 mV. In vitro biological characterization results indicated that Pluronic-PEI-DR5-TAT/DNA complexes had a higher specificity for the DR5 receptor and were taken up more efficiently by tumor cells than normal cells, compared to complexes formed with PEI 25 kDa or Pluronic-PEI. Thus, the novel complexes showed much lower cytotoxicity to normal cells and higher gene transfection efficiency in tumor cells than that exhibited by PEI 25 kDa and Pluronic-PEI. In summary, our novel, degradable non-viral tumor-targeting vector is a promising candidate for use in gene therapy.

## Background

In recent years, gene therapy has been considered to be the most promising strategy for the treatment of unresectable cancer. The vital technology for the success of gene therapy is gene delivery, which is greatly limited by the lack of a safe and efficient delivery system [1, 2]. Therefore, there is a critical need to develop a novel gene delivery system that can fulfill the special requirements for successful gene delivery, such as high transfection efficiency and low cytotoxicity as well as high level of targeting specificity to cancer cells. Over the past few decades, non-viral gene vectors, including cationic polymer, have attracted much attention due to their non-immunogenicity, structural diversity, and ease of production as compared to viral vectors [3–7]. Polyethylenimine (PEI) is a promising candidate among polycationic polymers used for transfection, which provides superior transfection efficiency due to its effective DNA condensation, uptake via the endocytosis pathway, and endosomal escape capacity [8–10]. However, there are still three outstanding issues to be solved if PEI is to be used as a gene carrier [11–14]. First and foremost, high-molecular-weight (HMW; ≥25 kDa) PEI has high transfection efficiency but also shows high cytotoxicity compared to low-molecular-weight (LMW; ≤2000 Da) PEI which displays lower cytotoxicity but limited delivering efficiency. Besides, PEI delivery relies simply on the electrostatic attraction between the positively charged polymer and negatively charged cells, which is non-selective. Last but not least, the stability of PEI/DNA complexes is improved as hydrophilicity increases, but this also reduces the ability to penetrate cellular membranes.

To overcome these limitations, considerable attempts have been made to modify PEI. Degradable PEIs have been formed by cross-linking with PEG/Pluronic chains that contain biodegradable moieties, and targeted vectors have been developed by combining PEI with ligands for cell- or tissue-specific targeting [15–20].

In our previous study, we developed a series of PEI-modified block polyplexes by grafting PEI with nonionic amphiphilic surfactant polyethers-Pluronic (Pluronic-PEI), which have been confirmed to enhance DNA condensation, cellular uptake, and transgene expression [21]. Moreover, the hydrophobic propylene oxide chain was thought to improve the biocompatibility of the vector, improve its stability, and prolong its circulation in blood in the same manner as PEGylation [13, 15, 16]. But the DNA/Pluronic-PEI complex could also be internalized into normal cells. To improve the targeting of the gene carrier for efficient cancer therapy, specific ligands which can bind to target cell-surface receptors, such as peptides, folate and antibodies, have been coupled to gene delivery vectors, which can trigger receptor-mediated endocytosis [22–24].

Receptor 1 (DR4) and receptor 2 (DR5) of tumor necrosis factor-α-related apoptosis-inducing ligand (TRAIL) are highly expressed on most tumor cells and thus present an ideal target for active targeting therapy [25–27]. A synthetic peptide, with the amino acid sequence YCKVILTHRCY, has been shown to bind specifically with DR5 [28, 29]. Furthermore, cell-penetrating peptides (CPPs) have been widely used in gene delivery systems to allow macromolecules to penetrate the cell membrane and target the cell nucleus. In addition, CPPs can enhance the transfection efficiency of PEI with low molecular weight [30–32]. The transduction domain of CPPs has been demonstrated to be an amino acid sequence of RKKRRQRRR (TAT) [33]. To take advantage of both of these peptides, we combined DR5-targeting peptide and linked it with TAT to obtain as a new multifunctional peptide (DR5-TAT) for use in our Pluronic-PEI vector. The multifunctional peptide is expected to not only target DR5 high expressed tumor cells but also promote the transmembrane ability and enhance transfection efficiency of the gene delivery system.

The overall preparation strategy for our new vector first involved preparation of DR5-TAT. Subsequently, this was coupled to Pluronic-PEI using cross-linking to prepare the final vector DR5-TAT-modified Pluronic-PEI (Pluronic-PEI-DR5-TAT), and after synthesis, its chemical and biophysical characterization was carried out. In addition, we prepared a model delivery system using reporter gene DNA (Pluronic-PEI-DR5-TAT/DNA) and evaluated its targeting effect, gene transfection efficiency, and safety in vitro. The results showed that the constructed novel gene delivery system has high non-viral transfection efficiency, high tumor cells, and tissue-specific targeting as well as good security and stability.

## Methods

### Synthesis of Pluronic-PEI and Pluronic-PEI-DR5-TAT

Pluronic-grafted PEI polyplexes (Pluronic-PEI) were synthesized as previously reported [21]. Briefly, PEI 2 kDa (0.20 g, 0.10 mmol) was dissolved in anhydrous dichloromethane (10 mL), and activated Pluronic (0.01 mmol) was separately dissolved in anhydrous ethanol (10 mL). PEI 2 kDa solution and Pluronic solution were then slowly added to anhydrous dichloromethane (10 mL) under constant stirring. The reaction was allowed to proceed at 25 °C overnight. After that, the reaction product was dialyzed against distilled water using a dialysis membrane (molecular weight cutoff (MWCO) 7 kDa; Spectrum Laboratories, Rancho Dominguez, CA, USA) at 4 °C for 48 h to remove byproducts. Finally, the purified product was lyophilized and stored at −20 °C for further use. The resulting sticky material was confirmed to be Pluronic-PEI by IR and ^1^H NMR spectroscopy.

**Fig. 1:**
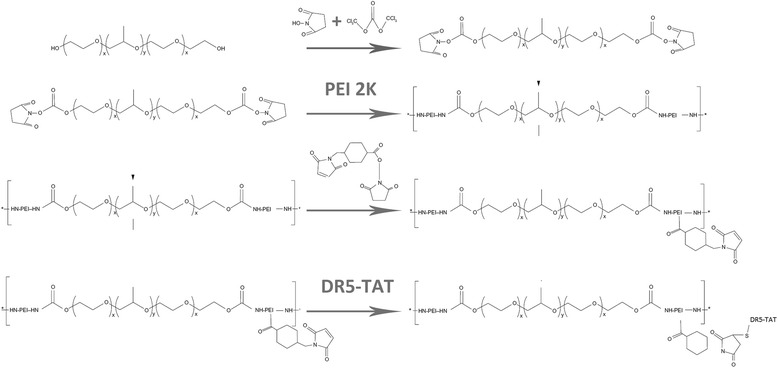
Synthetic scheme showing the preparation of Pluronic-PEI-DR5-TAT

To obtain DR5-TAT-modified Pluronic-PEI (Pluronic-PEI-DR5-TAT), 0.167 g *N*-succinimidyl-4-(*N*-maleimido-methyl)cyclo-hexane-1-carboxylate (SMCC) was dissolved in dimethyl sulfoxide (50 mL; 3.33 mg/mL), and Pluronic-PEI (0.452 g) were dissolved in phosphate-buffered saline (PBS; 50 mL; 0.1 M 9 mg/mL) [18]. Then, SMCC solution was added dropwise to Pluronic-PEI solution (SMCC:Pluronic-PEI = 2:1, mol/mol) with gentle stirring, and the mixture was further incubated for 40 min at 25 °C to obtain the maleimide-activated Pluronic-PEI. After that, the DR5-TAT PBS solution (10 mg/mL) was reacted with the maleimide-activated Pluronic-PEI solution (DR5-TAT: maleimide-activated Pluronic-PE = 2:1, mol/mol). The reaction mixture was stirred in the dark at 4 °C for 24 h. Finally, the reaction mixture was dialyzed (MWCO 7 kDa, Spectrum Laboratories) against distilled water for 24 h and lyophilized. After lyophilization, Pluronic-PEI-DR5-TAT, a solid white product, was obtained (Fig. 1). The identity of the material was confirmed using IR and ^1^H NMR spectroscopy.

The control vectors Pluronic-PEI-DR5 and Pluronic-PEI-TAT were synthesized in the same way as mentioned above.

### Characterizations of Pluronic-PEI and Pluronic-PEI-DR5-TAT

The ^1^H NMR spectra of Pluronic-PEI and Pluronic-PEI-DR5-TAT were recorded on a Mercury Plus 300 MHz spectrometer (Varian, USA) using approximately 10 mg sample dissolved in 0.6 mL deuterium oxide (D_2_O) at 25 °C. The IR spectra of Pluronic-PEI and Pluronic-PEI-DR5-TAT were measured using a Fourier transform IR (FT-IR) spectrometer (Varian, USA). KBr pellets of Pluronic-PEI and Pluronic-PEI-DR5-TAT were used in the IR instrument. The FT-IR spectra were acquired after 16 scans at 4000–400 cm^−1^.

### Degradation of Pluronic-PEI-DR5-TAT

The degradability of Pluronic-PEI-DR5-TAT was investigated under simulated in vivo conditions. Briefly, seven samples of Pluronic-PEI-DR5-TAT were prepared (0.5 g in 10 mL Dulbecco’s Modified Eagle’s medium (DMEM)) in centrifuge tubes. Then, tubes were incubated at 37 °C and shaken at 100 rpm. At specified periods (ranging from 5 to 60 h), a sample was lyophilized and the molecular weight of the lyophilized material was determined using gel permeation chromatography and multi-angle laser light scattering (laser wavelength, 690 nm).

### Preparation of Pluronic-PEI-DR5-TAT/DNA Complexes

Pluronic-PEI-DR5-TAT/DNA complexes were formed at predetermined N/P (nitrogen in cationic polymer to phosphate in nucleic acid) ratios. A mass per charge of 43 for PEI and a mass per phosphate of 325 Da for DNA were used to calculate the N/P ratio. An appropriate amount of DNA (10 μg) was added into various amounts of Pluronic-PEI-DR5-TAT solution, corresponding to N/P ratios ranging from 1:1 to 25:1, into a final volume of 300 μL. The solution was gently vortexed for 30 s and further incubated at 25 °C for 30 min.

### Gel Retardation Assay of Complexes

Electrophoresis was performed to investigate the DNA condensation ability of various amounts of polymer. Pluronic-PEI-DR5-TAT/DNA complexes were prepared at various N/P ratios from 1:1 to 8:1 in a final volume. The samples were electrophoresed on 1.0 % (*w*/*v*) agarose gels with ethidium bromide (0.5 mg/mL) and ran with Tris buffer for about 30 min at 100 V. DNA bands were visualized using a UV (254 nm) illuminator.

### Particle Size, Zeta Potential, and Morphology of Complexes

The mean particle size and zeta potential of the Pluronic-PEI-DR5-TAT/DNA complexes were analyzed at 25 °C using laser light scattering (Zetasizer ZS90; Malvern Instruments, Malvern, UK). Prior to measurement, Pluronic-PEI-DR5-TAT/DNA complexes with N/P ratios ranging from 1:1 to 25:1 were prepared as described above. The surface morphology of Pluronic-PEI/DNA and Pluronic-PEI-DR5-TAT/DNA samples (at N/P ratio of 15:1) was also observed under a transmission electron microscope (TEM; H600; Hitachi, Tokyo, Japan).

### DNase I Protection and Release Assay of Complexes

A DNase I protection assay was performed to evaluate the ability of Pluronic-PEI-DR5-TAT to protect DNA in complexes. Each solution of Pluronic-PEI-DR5-TAT/DNA complexes (at N/P ratio of 15:1) was divided equally and incubated at 37 °C for 1 h with DNase I at various concentrations of 0, 0.15, 0.75, 1.5, 2.25, 3.75, 4.5, 5.25, 6, 6.75, and 7.5 U DNase I/μg DNA. All of the samples were treated with 250 mM EDTA (10 μL) for 10 min to inactivate DNase I. Subsequently, the samples were further mixed with 10 μL heparin sodium (250 U/mL) and incubated at 25 °C for 120 min to displace DNA. Finally, agarose gel electrophoresis was conducted to analyze the extent of DNase I degradation in each sample.

PBS buffer solution containing dithiothreitol (DTT; 10 μL) was added to Pluronic-PEI-DR5-TAT/DNA solution to give a final concentration of 10 mM DTT, and the dispersions were incubated for 60 min. A control using PBS alone was also prepared. The samples were then assessed by agarose gel electrophoresis as described above.

### In vitro Cytotoxicity Evaluation

The cytotoxicity of polymers was evaluated in HeLa, HepG2, and NIH 3T3 cells (5 × 10^3^cells/well), which were cultured in 96-well plates for 18–24 h to achieve 80 % confluency before treatment. Various carrier materials (Pluronic-PEI-DR5-TAT, Pluronic-PEI, PEI 25 kDa, and PEI 2 K) at concentrations ranging from 5 to 160 μg/mL were added and incubated for another 24 h. Then, MTT reagent (20 μL; 5 mg/mL) was added to each well followed by DMSO (150 μL) 4 h later to dissolve the formazan crystals. Subsequently, the absorbance was measured at 570 nm using a microplate reader to assess the metabolic activity of the cells. The viability of untreated cells was set at 100 % (*A*_control_), and the absorbance of wells with medium and without cells was set as zero. The relative cell viability (%) compared to control cells was calculated using the formula *A*_test_/*A*_control_ × 100 %.

### Cellular Uptake and Competition Assay

DyLight-633 was used as a molecular probe to label PEI 25 kDa, Pluronic-PEI, and Pluronic-PEI-DR5-TAT. HeLa, HepG2, and NIH 3T3 cells (5 × 10^5^cells/well) were cultured in six-well plates for 8–12 h to reach about 80 % confluency before treatment. Labeled carrier materials at various concentrations (0, 0.20, 0.40, and 0.80 μM) were added, and the cells were incubated for 60 min. The cells were then washed three times with PBS to remove surface-associated complexes and were then trypsinized and resuspended in the medium. The efficiency of cell uptake was analyzed using a FACScan flow cytometer (Becton Dickinson, San Jose, CA, USA). Untreated cells were used as negative controls.

A competition assay was carried out to investigate DR5-mediated uptake of Pluronic-PEI-DR5-TAT/DNA complexes. Briefly, HeLa, HepG2, and NIH 3T3 cells were preincubated with 20 mM DR5 for 15 min; cells were then treated with Pluronic-PEI/DNA-cy3 and Pluronic-PEI-DR5-TAT/DNA-cy3 in a final concentration of 1 mM for 30 min at 37 °C. Cells treated with Pluronic-PEI/DNA-cy3 and Pluronic-PEI-DR5-TAT/DNA-cy3 without any inhibition was used as the control. Finally, the cells were washed three times with PBS and visualized under an Olympus I71-22/FL/PH inverted fluorescent microscope (Olympus Corporation, Tokyo, Japan). For quantitative analysis, HeLa, HepG2, and NIH 3T3 cells were treated as described above and permeabilized with cell lysis buffer (100 mL) before luciferase activity was measured in terms of relative light units (RLU) according to the manufacturer’s instructions (Promega). RLU was normalized against protein concentration in the cell extracts, which was determined using a Micro-BCA protein assay kit.

### Transfection Efficiency Assay

A green fluorescent protein (pEGFP-N2) plasmid was used as a reporter gene to examine the ability of Pluronic-PEI-DR5-TAT to transfect HeLa and HepG2 cells. Cells (1 × 10^5^ cells/well) were seeded in 24-well plates and incubated for 18–24 h prior to the transfection experiments. Different polymer/DNA complexes (containing 3 μg pGL3) with various N/P ratios ranging from 1:1 to 15:1 were added to the cells and incubated for 4 h at 37 °C under 5 % CO_2_ atm. After that, the medium was replaced with fresh, serum-containing complete media, and the cells were incubated for a further 48 h. Finally, the cells were washed three times with PBS and visualized under a fluorescent microscope.

A pGL3 plasmid control was also employed to examine the gene delivery efficiency of polymer. The cells were treated as described above, and plasmid DNA (pEGFP-N2; 3 μg per well) was applied. After incubation for 48 h, fluorescence quantitative analysis was carried out as described in the competition assay section.

## Results and Discussion

### Synthesis and Characterization of Pluronic-PEI-DR5-TAT

The preparation of Pluronic-PEI-DR5-TAT began when free hydroxyl groups on the Pluronic polyether chains were activated using succinimidyl carbonate and then linked to the amino groups of to give Pluronic-PEI [21]. Next, SMCC was used as a cross-linker between the remaining amino groups of Pluronic-PEI and the sulfhydryl group on the multifunctional peptide DR5-TAT to obtain Pluronic-PEI-DR5-TAT, as shown in Fig. 1.

**Fig. 2:**
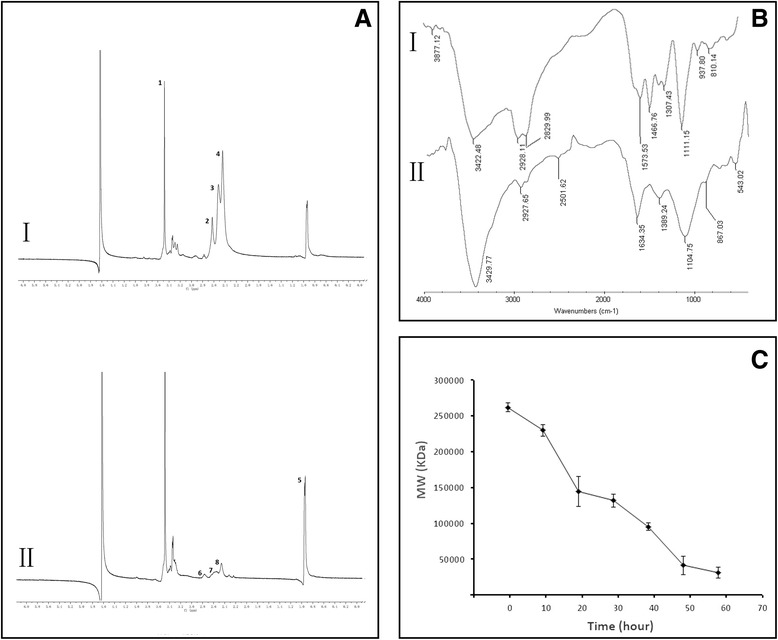
**a** ^1^H NMR spectra of (*I*) Pluronic-PEI and (*II*) Pluronic-PEI-DR5-TAT. **b** IR spectra of (*I*) Pluronic-PEI and (*II*) Pluronic-PEI-DR5-TAT. **c** Degradation of Pluronic-PEI-DR5-TAT after incubation with buffer at 37 °C. Molecular weight (MW) was measured by gel permeation chromatography with multi-angle laser light scattering (*n* = 3)

**Fig. 3:**
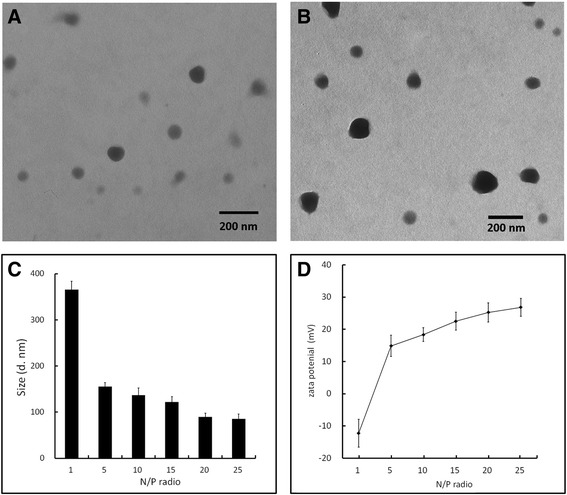
Particle size, zeta potential, and morphology of Pluronic-PEI-DR5-TAT/DNA complex. **a** Morphology of Pluronic-PEI/DNA complexes at N/P ratio of 15. **b** Morphology of Pluronic-PEI-DR5-TAT/DNA at N/P ratio of 15. **c** Average particle sizes (nm) of Pluronic-PEI-DR5-TAT/DNA complexes at various N/P ratios (*n* = 3). **d** Average zeta potential (mV) of Pluronic-PEI-DR5-TAT/DNA complexes with various N/P ratios (*n* = 3)

**Fig. 4:**
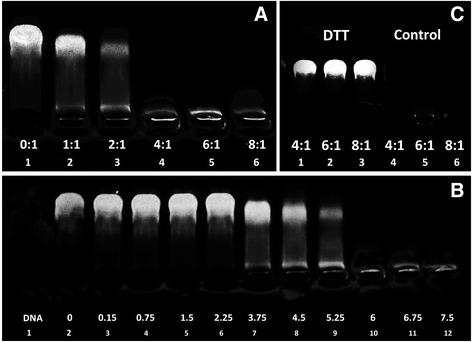
**a** Agarose gel electrophoresis showing retardation of pGL3 plasmid DNA by Pluronic-PEI-DR5-TAT at different N/P ratios, 0:1 (DNA only), 1:1, 2:1, 4:1, 6:1, and 8:1. **b** Pluronic-PEI-DR5-TAT protects plasmid DNA DNase I-mediated degradation at various concentrations was estimated by agarose gel electrophoresis. Naked DNA was used as control. **c** Influence of dithiothreitol on Pluronic-PEI-DR5-TAT/DNA complexes at various N/P ratios (4:1, 6:1, 8:1). The *last three lanes* were treated with 10 mM DTT

The ^1^H NMR spectrum (Fig. 2a(I)) showed –C*H*_2_C*H*_2_O– proton peaks at 3.5 ppm (Fig. 2a(I-1)) and –C*H*_2_C*H*_2_N*H*– protons peaks at δ2.4–3.6 ppm (Fig. 2a(I-2, 3, 4)), which were the characteristic signal of Pluronic-PEI as conformed in our prior report [24]. Fig. 2a(II) shows the ^1^H NMR spectrum of Pluronic-PEI-DR5-TAT, in which the integral of the signal is at 0.9 ppm (Fig. 2a(II-5)), increased and the signals at 2.4–2.6 ppm (Fig. 2a(II-678)) decreased. Furthermore, proton peaks moved upfield compared to those in Fig. 2a(I) owing to the electronic screening effect resulting from DR5-TAT attachment. These results indicate that DR5-TAT was successfully linked to Pluronic-PEI.

The identity of Pluronic-PEI-DR5-TAT was also confirmed by FT-IR. As shown in Fig. 2b, compared with the IR spectra of Pluronic-PEI, a phenyl signal appeared at 1634 cm^−1^ in Pluronic-PEI-DR5-TAT spectrum due to the phenyl group of DR5-TAT. This further confirmed the successful coupling of DR5-TAT to Pluronic-PEI.

### Degradation of Polyplexes

The degradability of gene vectors is important with respect to safety*.* Non-degradable PEI vectors such as PEI 25 kDa may accumulate in vivo and cause potential cytotoxicity due to the lack of degradation or excretion pathways. It is therefore expected that the amide bonds in Pluronic-PEI-DR5-TAT are susceptible to hydrolysis leading to the generation of poloxamer oligomers and low molecular weight PEI under physiological conditions and resulting in low cytotoxicity [21]. The in vitro degradation study (Fig. 2c) indicated that Pluronic-PEI-DR5-TAT could be degraded slowly under simulated in vivo conditions, and complete degradation took about 60 h, indicating that Pluronic-PEI-DR5-TAT could ensure transfection efficiency while reducing the toxic effect on cells.

To analyze the degradation kinetics of Pluronic-PEI-DR5-TAT, the degradation curve was fitted to three different kinetic models (Table 1). As shown in Table 1, the degradation profile of Pluronic-PEI-DR5-TAT could be well described by the first-order kinetics model. Based on the model, the half-life of Pluronic-PEI-DR5-TAT is about 30.7 h.

**Table 1:** Fitting of degradation degree of Pluronic-PEI-DR5-TAT to different kinetic models

Model	Equation	*R*	Mse
Zero-order kinetic	Q = 0.0125t + 0.1157	0.9743	0.01123
First-order kinetic	ln(1 − Q) = −0.285t + 0.0213	0.9968	0.00056
Higuchi	Q = 0.1058t^1/2^ − 0.0047	0.9959	0.00108

### Particle Size, Zeta Potential, and Morphology of Complexes

Complex formation relies on a self-assembly process between negatively charged plasmid DNA and positively charged polymer [12]; therefore, the ability of a cationic polymer to condense DNA is a prerequisite for effective gene delivery. Particle size also affects gene delivery as complexes with smaller sizes tend to accumulate in tumors due to the enhanced permeation and retention (EPR) effect and internalize into cells more quickly [34]. A positive surface charge is also required for the complex to be able to bind to negatively charged cell surfaces and facilitate cellular uptake.

The particle size and zeta potential of Pluronic-PEI-DR5-TAT/DNA complexes were determined at various N/P ratios using dynamic light scattering, and nanoparticles morphology was observed using TEM. The results are shown in Fig. 3a–d. As shown in Fig. 3a, b, the morphology of Pluronic-PEI-DR5-TAT/DNA complexes was roughly spherical, which is similar to the Pluronic-PEI/DNA complexes. However, compared to Pluronic-PEI/DNA complexes, there was an increase in particle size of about 28 nm, presumably owing to the presence of DR5-TAT. The particle size of the complexes decreased significantly as N/P ratio increased, which indicated that condensation capability of polymers is strengthened as charge increases (Fig. 3c). When the N/P ratio was at 15, the particle size and the zeta potential of Pluronic-PEI-DR5-TAT/DNA complexes were about 122 ± 11.6 nm and 22 ± 2.8 mV (Fig. 3c, d), respectively, which is very convenient for cell uptake. Moreover, all of the polymer/DNA complexes (except Pluronic-PEI-DR5-TAT at the N/P ratio of 1) were positively charged (Fig. 3d). As N/P ratio increased from 1 to 25, the positive charge of the complexes also increased, which is consistent with the following results of gel retardation assay.

### DNase I Protection and Release Assay of Complexes

Protecting DNA from nuclease-mediated degradation is the primary responsibility of any gene delivery carrier [35]. The DNA condensation capability of Pluronic-PEI-DR5-TAT was measured by gel retardation assay. As shown in Fig. 4a, the migration of the DNA plasmid was increasingly retarded as the amount of the Pluronic-PEI-DR5-TAT was increased. Migration was completely retarded at an N/P ratio of 4:1, confirming that Pluronic-PEI-DR5-TAT binds to DNA and neutralizes its charge.

As shown in Fig. 4b, after the complexes solution were incubated with different concentration of DNase I, DNA was still protected from digestion by DNase I (lanes 2–8), even at a concentration of 5.25 U DNase I/μg DNA (lane 8), while naked DNA was degraded by DNase I at a concentration of 0.08 U DNase I/μg DNA (lane 1), which is consistent with the literature [36].

Figure 4c shows the release property of DNA from complexes in the intracellular environment. DTT was used to simulate the intracellular environment in vivo, mediating the ester bond of Pluronic-PEI-DR5-TAT fission and testing the DNA release property of Pluronic-PEI-DR5-TAT (lanes 1–3). The vector without DTT was used as control group (lanes 4–6). DNA release from Pluronic-PEI-DR5-TAT was observed in the presence of 5.0 mM DTT, which simulated the 0.1–10 mM glutathione found in vivo (lanes 1–3), compared to the control group which showed completely retard plasmid DNA migration (lanes 4–6). In contrast, there was almost no DNA released from PEI 25 kDa/DNA complexes when treated with DTT [37]. This result indicates that Pluronic-PEI-DR5-TAT could dissociate easily in the intracellular environment, which was similar to the results observed in our previous work, which showed that Pluronic-PEI dissociation was most likely because DTT mediated ester bond fission, leading to more DNA to release and enhancing gene expression.

### Cytotoxicity of Polymers In vitro

**Fig. 5:**
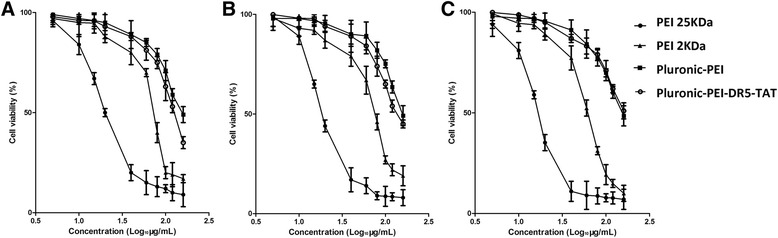
Cytotoxicity of polyplexes at various concentrations in **a** HeLa, **b** HepG2, and **c** NIH 3T3 cells. The cells were incubated using different polyplexes for a time period of 24 h (mean ± SD, *n* = 6)

The cytotoxicity of HMW PEI is largely due to the high density of amino groups. Pluronic-PEI is expected to have lower cytotoxicity than HMW PEI with similar molecular weight (25 kDa) [24]. The cytotoxicity of Pluronic-PEI-DR5-TAT was measured in three cell lines (HeLa, HepG2, and NIH 3T3) and compared to PEI 2 kDa, PEI 25 kDa, and Pluronic-PEI using the MTT assay. The half maximal inhibitory concentration (IC_50_) curves are shown in Fig. 5. The IC_50_ values of the carrier materials (PEI 2 kDa, PEI 25 kDa, Pluronic-PEI, and Pluronic-PEI-DR5-TAT) ranged from 16.32 to 294.7 μg/mL (HeLa: 71.28, 17.24, 294.7, 286.9 μg/mL; HepG2: 69.22, 18.6, 217.1, 189.6 μg/ mL; NIH 3T3: 60.83, 16.32, 606.6, 938.5 μg/mL). Among considerable prior attempts have been made to modify PEI to reduce its cytotoxicity, the most effective one is an introduction of phospholipid-modified polyethylenimine [15, 20, 38, 39]; its IC_50_ value is about 123 μg/mL. As shown in Fig. 5, PEI 25 kDa showed clear dose-dependent cytotoxicity effects, and most of the treated cells were killed at a concentration of 20 μg/mL. The cell viabilities after treatment with Pluronic-PEI or Pluronic-PEI-DR5-TAT were more than 80 %, even up to a concentration of 60 μg/mL. This indicated that they are suitable for gene delivery even at a high concentration. The low cytotoxicity of Pluronic-PEI and Pluronic-PEI-DR5-TAT may be the result of the lower amino group density compared to HMW PEI. Moreover, the IC_50_ values of Pluronic-PEI-DR5-TAT in tumor cells (HeLa and HepG2) were significantly higher than in normal cells (NIH 3T3); in other words, cells which expressed high levels of DR5 were more sensitive to Pluronic-PEI-DR5-TAT than the DR5-negative NIH 3T3 cells. This suggests that the binding ability and specificity of Pluronic-PEI-DR5-TAT to DR5 reduces toxicity in normal cells. These results indicated that Pluronic-PEI-DR5-TAT is an ideal vector for gene transfection because its low toxicity to normal cells will allow a higher dose of Pluronic-PEI-DR5-TAT to be used.

### Cellular Uptake and Competition Assay

**Fig. 6:**
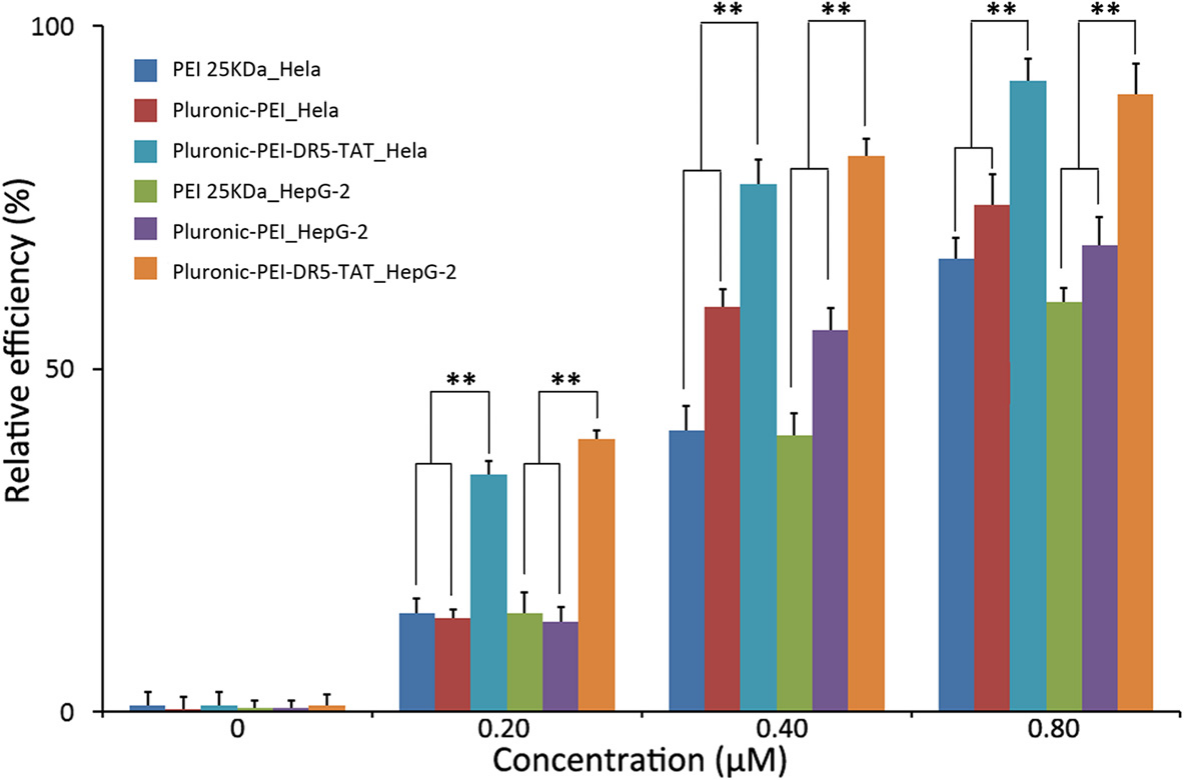
Flow cytometry study of cellular uptake of polyplexes. Total fluorescence intensity (*n* = 3, error bars represent standard deviation) after a 60-min incubation of DyLight-633-labeled PEI 2 kDa, Pluronic-PEI, and Pluronic-PEI-DR5-TAT at different concentrations (0, 0.20, 0.40, 0.80 μM) in HeLa and HepG2 cells. Significance: **P < 0.01

Efficient entry of complexes into cells is a critical step for gene transcription. Many factors, such as particle size, zeta potential, and ligand-receptor interaction, have a huge effect on cell uptake [36]. HeLa and HepG2 cells were used as model tumor cells to investigate the in vitro cellular uptake of Pluronic-PEI-DR5-TAT. As shown in Fig. 6, at the same concentrations, PEI 25 kDa and Pluronic-PEI had similar cell uptake efficiency in HeLa and HepG2 cells, but Pluronic-PEI-DR5-TAT had significantly higher cell uptake efficiency than both. Our results suggest that DR5-TAT modification of Pluronic-PEI enhances transfer across the cell membrane and increases the internalization of Pluronic-PEI-DR5-TAT owing to specific ligand-receptor binding, DR5-mediated endocytosis mediated, and the enhanced cell penetration due to TAT.

**Fig. 7:**
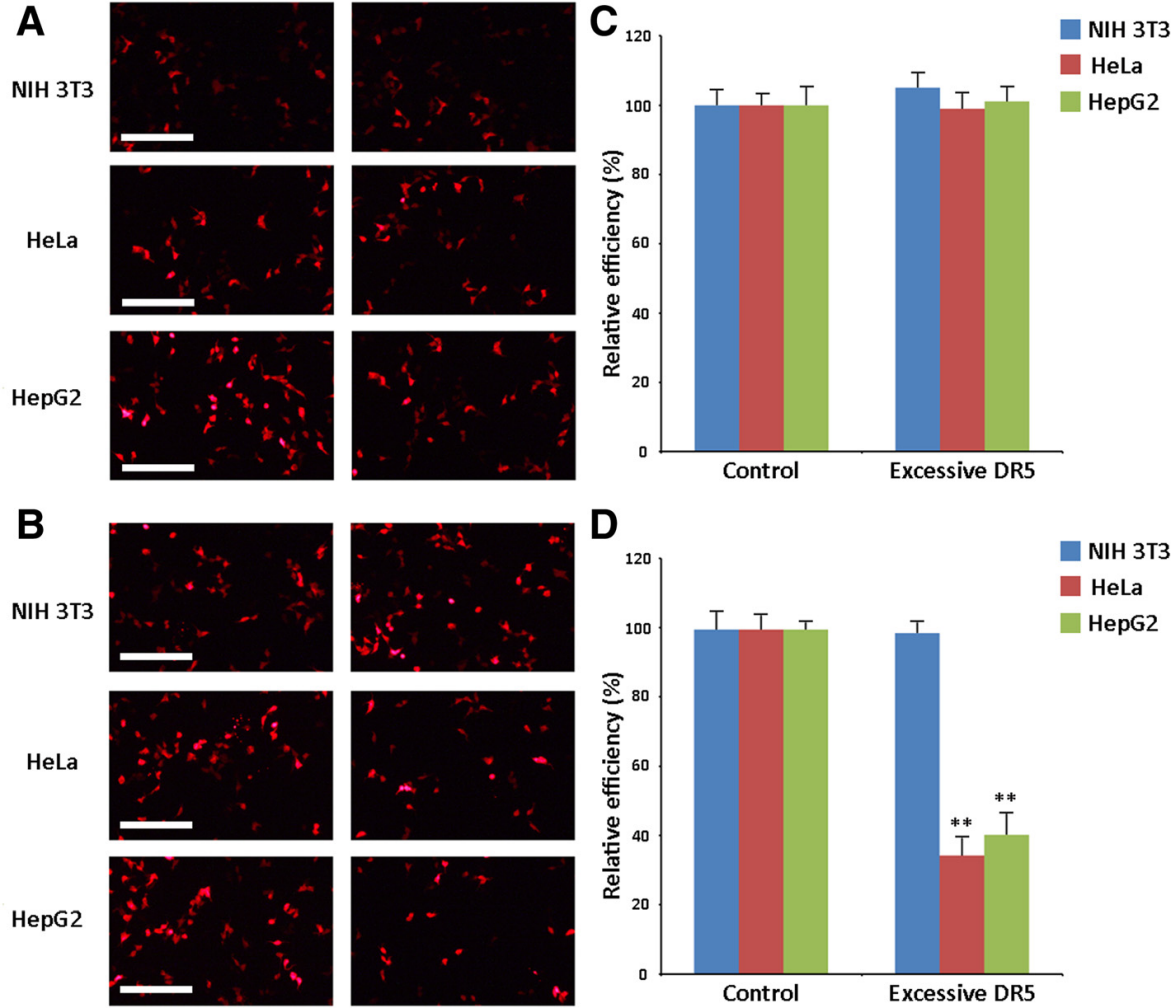
Competition inhibition assay of polyplexes. **a** The cellular uptake of Pluronic-PEI/DNA-cy-3 complexes in HeLa, HepG2, and NIH 3T3 cells pretreated with DR5 was examined by fluorescence microscopy (*scale bar*: 200 μm). **b** Relative uptake efficiency of different polyplexes after treatment with inhibitor (DR5) in cells. Cellular uptake without any inhibition was used as control (mean ± SD, *n* =3). **c** The cellular uptake of Pluronic-PEI-DR5-TAT/DNA-cy3 complexes in HeLa, HepG2, and NIH 3T3 cells pretreated with DR5 was examined by fluorescence microscopy (*scale bar*: 200 μm). **d** Relative uptake efficiency of different polyplexes after treatment with inhibitor (DR5) in cells. Cellular uptake without any inhibition was used as control (mean ± SD, *n* = 3). Significance: **P < 0.01

To confirm the specificity of the interaction between Pluronic-PEI-DR5-TAT/DNA complexes and the targeted cells, a competition assay was performed using free DR5. The uptake efficiency of Pluronic-PEI-DR5-TAT/DNA-cy3 and Pluronic-PEI/DNA-cy3 complexes (N/P ratio of 15) was investigated using DR5 as a competitor. The results were observed by fluorescence microscopy (Fig. 7a, b), and the fluorescence intensity were quantitative determined (Fig. 7c, d). The cell uptake efficiency of Pluronic-PEI-DR5-TAT/DNA complexes in HepG2 and HeLa cells pretreated with DR5-TAT was significantly inhibited compared with untreated cells (Fig. 7b, d), whereas that of Pluronic-PEI/DNA complexes was only slightly changed or unchanged (Fig. 7a, c). It is worth mentioning that the cellular uptake efficiency of neither Pluronic-PEI-DR5-TAT/DNA complexes nor Pluronic-PEI/DNA complexes in NIH 3T3 cells (DR5-negative) was affected by DR5. Therefore, the availability of DR5 was essential for the entry of Pluronic-PEI-DR5-TAT/DNA complexes into tumor cells through a receptor-mediated delivery system.

### Transfection Efficiency

**Fig. 8:**
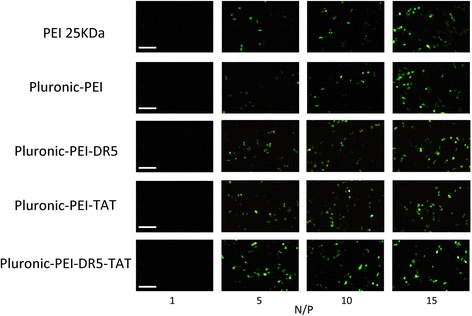
Fluorescence microscopy images (*scale bar*: 200 μm) of HeLa cells after transfection with pEGFP-N2/PEI 25 kDa, pEGFP-N2/Pluronic-PEI or pEGFP-N2/Pluronic-PEI-DR5, pEGFP-N2/Pluronic-PEI-TAT, and pEGFP-N2/Pluronic-PEI-DR5-TAT at different N/P ratios

**Fig. 9:**
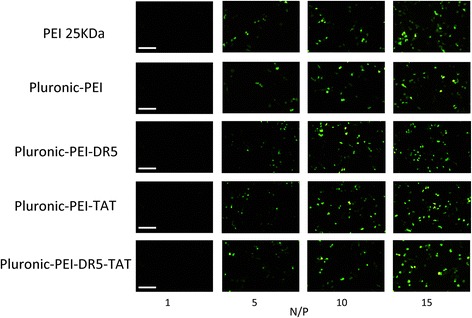
Fluorescence microscopy images (*scale bar*: 200 μm) of HepG2 cells after transfection with pEGFP-N2/PEI 25 kDa, pEGFP-N2/Pluronic-PEI or pEGFP-N2/Pluronic-PEI-DR5, pEGFP-N2/Pluronic-PEI-TAT, and pEGFP-N2/Pluronic-PEI-DR5-TAT at different N/P ratios

**Fig. 10:**
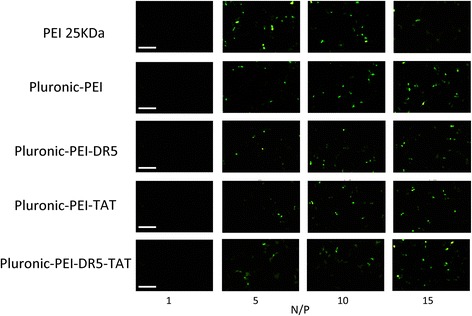
Fluorescence microscopy images (*scale bar*: 200 μm) of NIH 3T3 cells after transfection with pEGFP-N2/PEI 25 kDa, pEGFP-N2/Pluronic-PEI or pEGFP-N2/ Pluronic-PEI-DR5, pEGFP-N2/Pluronic-PEI-TAT, and pEGFP-N2/Pluronic-PEI-DR5-TAT at different N/P ratios

The transfection efficiency of polymer/pEGFP-N2 (at various N/P ratios) in HeLa, HepG2, and NIH 3T3 cells was observed by fluorescence microscopy. Figures 8, 9, and 10 show that gene transfection efficiencies of all five polymers (PEI 25 kDa, Pluronic-PEI, Pluronic-PEI-DR5, Pluronic-PEI-TAT, and Pluronic-PEI-DR5-TAT) all increased in line with N/P ratio in all five cell lines. The pEGFP reporter gene expression in HeLa and HepG2 cells treated with Pluronic-PEI-DR5-TAT complexes was higher than PEI 25 kDa, Pluronic-PEI complexes, Pluronic-PEI-DR5, or Pluronic-PEI-TAT at all N/P ratios (Figs. 8 and 9). No obvious differences were observed between the same five polymer/DNA complexes in NIH 3T3 cells (Fig. 10).

**Fig. 11:**
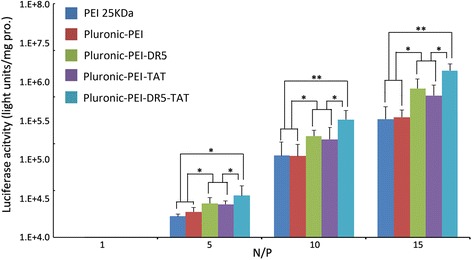
Luciferase activity analysis (mean ± SD, *n* = 4) after transfection of HeLa with pGL-3/PEI 25 kDa, pGL-3/Pluronic-PEI, pGL-3/Pluronic-PEI-DR5, pGL-3/Pluronic-PEI-TAT, or pGL-3/Pluronic-PEI-DR5-TAT at different N/P ratios. *P < 0.05, **P < 0.01

**Fig. 12:**
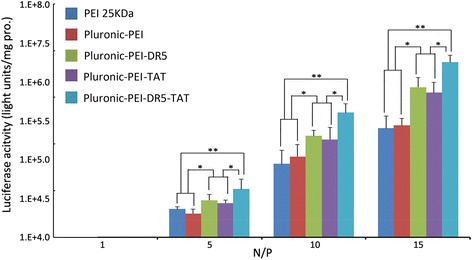
Luciferase activity analysis (mean ± SD, *n* = 4) after transfection of HepG2, with pGL-3/PEI 25 kDa, pGL-3/Pluronic-PEI, pGL-3/Pluronic-PEI-DR5, pGL-3/Pluronic-PEI-TAT, or pGL-3/Pluronic-PEI-DR5-TAT at different N/P ratios. *P < 0.05, **P < 0.01

**Fig. 13:**
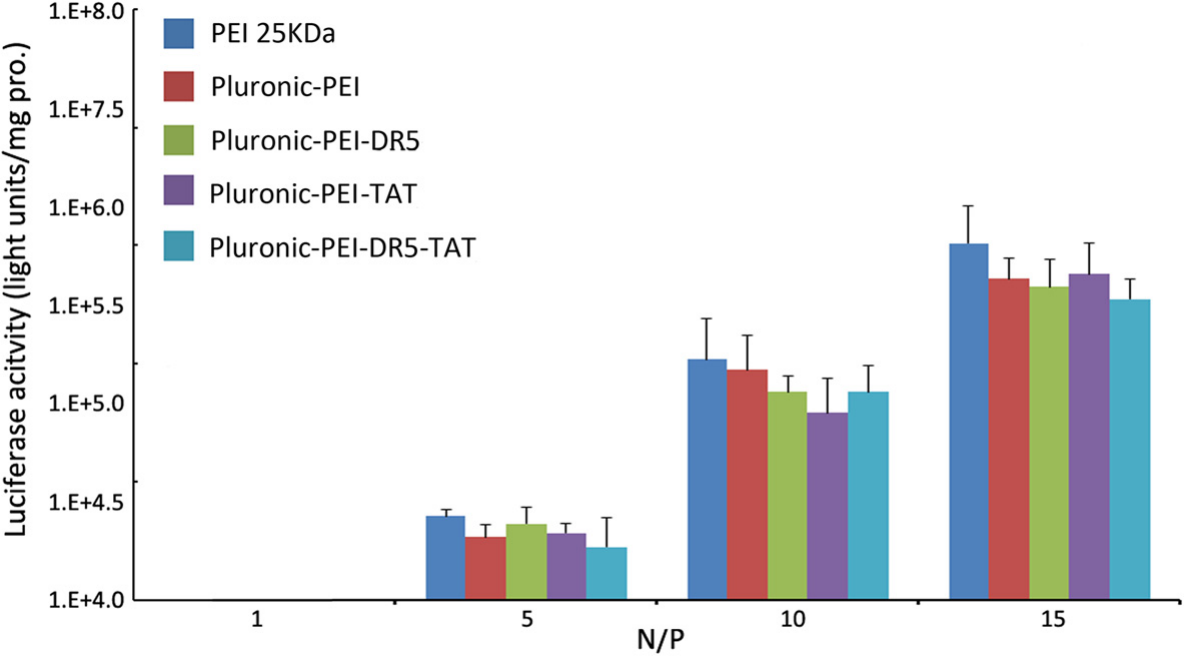
Luciferase activity analysis (mean ± SD, *n* = 4) after transfection of NIH 3T3 cells with pGL-3/PEI 25 kDa, pGL-3/Pluronic-PEI, pGL-3/Pluronic-PEI-DR5, pGL-3/Pluronic-PEI-TAT, or pGL-3/Pluronic-PEI-DR5-TAT at different N/P ratios

To evaluate the transfection efficiency mediated by Pluronic-PEI-DR5-TAT, a luciferase assay was also performed in HeLa, HepG2, and NIH 3T3 cells, and the results are shown in Fig. 11. The complexes possessed the highest transfection efficiency at an N/P ratio of 15. The transfection efficiency of Pluronic-PEI-DR5-TAT was shown to be significantly higher than that of Pluronic-PEI-DR5 or Pluronic-PEI-TAT (*P < 0.05) and much significantly higher than that of PEI 25 kDa or Pluronic-PEI in HeLa and HepG2 cells (**P < 0.01), while PEI 25 kDa and Pluronic-PEI demonstrated similar transfection efficiency (Figs. 11 and 12). In HeLa cells at an N/P ratio of 15, the luciferase expression induced by transfection with Pluronic-PEI-DR5-TAT/pGL3-control complexes was 1.92 and 1.30 times more than that induced by Pluronic-PEI-DR5 or Pluronic-PEI-TAT, 5.5 and 5.9 times more than that induced by Pluronic-PEI or PEI 25 kDa, respectively. In HepG2 cells, Pluronic-PEI-DR5-TAT/pGL3-control complexes induced 2.50 and 1.21 times more than that induced by Pluronic-PEI-DR5 or Pluronic-PEI-TAT, 10.1 and 11.2 times more luciferase expression than Pluronic-PEI or PEI 25 kDa, respectively. There was no significant difference in the luciferase expression induced by the three vectors in NIH 3T3 cells (Fig. 13). Since there is over-expression of DR5 on HeLa and HepG2 cells, the multifunctional DR5-TAT may also promote cargo transport via receptor-mediated endocytosis [16]. Moreover, the TAT peptide could increase membrane penetration, and it is the combined effect of DR5 and TAT that improves the gene transport ability of DR5-TAT-modified Pluronic-PEI. These results indicate that Pluronic-PEI conjugated with DR5-TAT peptide has excellent gene transfection efficiency and a high level of targeting specificity to DR5-positive cells.

## Conclusions

A novel non-viral gene vector, Pluronic-PEI-DR5-TAT, was successfully constructed by cross-linking LMW-PEI with Pluronic and further coupled a multifunctional peptide DR5-TAT to form the copolymer for targeting DR5-positive cancer cells and increasing cellular uptake efficiency, which is believed to increase gene transfection efficiency and reduce toxicity to normal cells. We have confirmed that Pluronic-PEI-DR5-TAT could easily form complexes with DNA and that it had appropriate biophysical characteristics for effective gene delivery. Moreover, the novel gene delivery system showed much lower cytotoxicity on DR5-negative NIH 3T3 cells, significantly higher cellular uptake efficiency and gene transfection efficiency than PEI 25 kDa and Pluronic-PEI on HeLa and HepG2 cells than over-expression of DR5. In summary, Pluronic-PEI-DR5-TAT is a potential DR5 selective targeting gene vector for cancer gene therapy.

## References

[1] Verma IM, Somia N (1997). Gene therapy promise, problems and prospects. Nature.

[2] Górecki DC (2001). Prospects and problems of gene therapy: an update. Expert Opin Emerg Dr.

[3] Ohlfest JR, Freese AB, Largaespada DA (2005). Nonviral vectors for cancer gene therapy: prospects for integrating vectors and combination therapies. Curr Gene Ther.

[4] Kim TI, Lee M, Kim SW (2010). A guanidinylated bioreducible polymer with high nuclear localization ability for gene delivery systems. Biomaterials.

[5] Song HP, Yang JY, Lo SL, Wang Y, Fan WM (2010). Gene transfer using self-assembled ternary complexes of cationic magnetic nanoparticles, plasmid DNA and cell-penetrating Tat peptide. Biomaterials.

[6] Kim NJ, Jiang DH, Jacobi AM, Lennox KA, Rose SD, Behlke MA (2012). Synthesis and characterization of mannosylated pegylated polyethylenimine as a carrier for siRNA. Int J Pharm.

[7] Pan S, Cao D, Yi W, Huang H, Feng M (2013). A biodegradable and serum-resistant gene delivery carrier composed of polyamidoamine-poly N, N’-di-(2-amino-ethyl) aminoethy glutamine copolymer. Colloid Surface B.

[8] Boussif O, Lezoualc’h F, Zanta MA, Mergny MD, Scherman D (1995). A versatile vector for gene and oligonucleotide transfer into cells in culture and in vivo: polyethylenimine. Proc Natl Acad Sci U S A.

[9] Jere D, Jiang HL, Arote R, Kim YK, Choi YJ, Cho MH (2009). Degradable polyethylenimines as DNA and small interfering RNA carriers. Expert Opin Drug Deliv.

[10] Schäfer J, Höbel S, Bakowsky U, Aigner A (2010). Liposome-polyethylenimine complexes for enhanced DNA and siRNA delivery. Biomaterials.

[11] Deng R, Yue Y, Jin F, Chen Y, Kung HF (2009). Revisit the complexation of PEI and DNA-how to make low cytotoxic and highly efficient PEI gene transfection non-viral vectors with a controllable chain length and structure. J Control Release.

[12] Huang HL, Yu H, Tang G, Wang Q, Li J (2010). Low molecular weight polyethylenimine cross-linked by 2-hydroxypropyl-gamma- cyclodextrin coupled to peptide targeting HER2 as a gene delivery vector. Biomaterials.

[13] Shi S, Guo QF, Kan B, Fu S, Wang X (2009). A novel Poly(ε-caprolactone)-Pluronic-Poly(ε-caprolactone) grafted Polyethyleneimine (PCFC-g-PEI), Part 1, synthesis, cytotoxicity, and in vitro transfection study. BMC Biotechnol.

[14] Park J, Singha K, Son S (2012). A review of RGD-functionalized nonviral gene delivery vectors for cancer therapy. Cancer Gene Ther.

[15] Park MR, Han KO, Cho MH, Nah JW, Choi YJ (2005). Degradable polyethylenimine-alt- poly(ethylene glycol) copolymers as novel gene carriers. J Control Release.

[16] Biswal BK, Debata NB, Verma RS (2010). Development of a targeted siRNA delivery system using FOL-PEG-PEI conjugate. Mol Biol Rep.

[17] Kang JH, Tachibana Y, Kamata W, Mahara A, Harada-Shiba M (2010). Liver-targeted siRNA delivery by polyethylenimine (PEI)-pullulan carrier. Bioorg Med Chem.

[18] Liu KH, Wang XY, Fan W (2012). Degradable polyethylenimine derivate coupled to a bifunctional peptide R13 as a new gene-delivery vector. Int J Nanomed.

[19] Xu ZH, Jin JF, Siu LK, Yao H, Sze J, Sun HZ (2012). Folic acid conjugated mPEG-PEI600 as an efficient non-viral vector for targeted nucleic acid delivery. Int J Pharm.

[20] Wang M, Wu B, Lu P, Tucker JD, Milazi S, Shah SN (2014). Pluronic-PEI copolymers enhance exon-skipping of 20-O-methyl phosphorothioate oligonucleotide in cell culture and dystrophic mdx mice. Gene Ther.

[21] Fan W, Wu X, Ding BY, Gao J, Cai Z (2012). Degradable gene delivery systems based on Pluronics-modified low-molecular-weight polyethylenimine: preparation, characterization, intracellular trafficking, and cellular distribution. Int J Nanomed.

[22] Cai LL, Liu P, Li X, Huang X, Ye YQ (2011). RGD peptide-mediated chitosan-based polymeric micelles targeting delivery for integrin-overexpressing tumor cells. Int J Nanomed.

[23] Hamano N, Negishi Y, Fujisawa A, Manandhar M, Sato H (2012). Modification of the C16Y peptide on nanoparticles is an effective approach to target endothelial and cancer cells via the integrin receptor. Int J Pharm.

[24] Xing HB, Pan HM, Fang Y, Zhou XY, Pan Q (2014). Construction of a tumor cell-targeting non-viral gene delivery vector with polyethylenimine modified with RGD sequence-containing peptide. Oncol Lett.

[25] Chuntharapai A, Dodge K, Grimmer K, Schroeder K, Marsters SA (2001). Isotype-dependent inhibition of tumor growth in vivo by monoclonal antibodies to death receptor 4. J Immunol.

[26] Cretney E, Takeda K, Smyth MJ (2007). Cancer: novel therapeutic strategies that exploit the TNF-related apoptosis-inducing ligand (TRAIL)/TRAIL receptor pathway. Int J Biochem Cell Biol.

[27] Ding B, Wu X, Fan W, Wu Z, Gao J (2011). Anti-DR5 monoclonal antibody-mediated DTIC-loaded nanoparticles combining chemotherapy and immunotherapy for malignant melanoma: target formulation development and in vitro anticancer activity. Int J Nanomed.

[28] Li B, Russell SJ, Compaan DM, Totpal K, Marsters SA (2006). Hymowitz and Sachdev S. Sidhu. Activation of the proapoptotic death receptor DR5 by oligomeric peptide and antibody agonists. J Mol Biol.

[29] Vrielink J, Heins MS, Setroikromo R, Szegezdi E, Mullally MM (2010). Synthetic constrained peptide selectively binds and antagonizes death receptor 5. FEBS J.

[30] Chen YJ, Liu BR, Dai YH, Lee CY, Chan MH (2012). A gene delivery system for insect cells mediated by arginine-rich cell-penetrating peptides. Gene.

[31] Bolhassani A (2011). Potential efficacy of cell-penetrating peptides for nucleic acid and drug delivery in cancer. Biochim Biophys Acta.

[32] Hu Y, Xu BH, Ji QX, Shou D, Sun X (2014). A mannosylated cell-penetrating peptide-graft-polyethylenimine as a gene delivery vector. Biomaterials.

[33] Santos-Cuevas CL, Ferro-Flores G, Arteaga de Murphy C, Ramírez Fde M, Luna-Gutiérrez MA (2009). Design, preparation, in vitro and in vivo evaluation of 99mTc-N2S2-Tat(49–57)-bombesin: a target-specific hybrid radiopharmaceutical. Int J Pharm.

[34] Maeda H, Wu J, Sawa T, Matsumura Y, Hori K (2000). Tumor vascular permeability and the EPR effect in macromolecular therapeutics: a review. J Control Release.

[35] Liang W, Gong H, Yin D, Lu S, Fu Q (2011). High-molecular-weight polyethyleneimine conjuncted pluronic for gene transfer agents. Chem Pharm Bull (Tokyo).

[36] Hao J, Sha X, Tang Y, Jiang Y, Zhang Z (2009). Enhanced transfection of polyplexes based on pluronic-polypropylenimine dendrimer for gene transfer. Arch Pharm Res.

[37] Piest M, Lin C, Mateos-Timoneda MA, Lok MC, Hennink WE (2008). Novel poly (amido amine)s with bioreducible disulfide linkages in their diamino-units: structure effects and in vitro gene transfer properties. J Control Release.

[38] Navarro G, Essex S, Sawant RR, Biswas S, Nagesha D, Sridhar S (2014). Phospholipid-modified polyethylenimine-based nanopreparations for siRNA-mediated gene silencing: implications for transfection and the role of lipid components. Nanomedicine.

[39] Aravindan L, Bicknell KA, Brooks G, Khutoryanskiy VV, Williams AC (2013). A comparison of thiolated and disulfide-crosslinked polyethylenimine for nonviral gene delivery. Macromol Biosci.

